# Collateral Impact of Public Health and Social Measures on Respiratory Virus Activity during the COVID-19 Pandemic 2020–2021

**DOI:** 10.3390/v14051071

**Published:** 2022-05-17

**Authors:** Chiara Achangwa, Huikyung Park, Sukhyun Ryu, Moo-Sik Lee

**Affiliations:** 1 Department of Preventive Medicine, College of Medicine, Konyang University, Daejeon 35365, Korea; ciaraacha@gmail.com (C.A.); phk981117@naver.com (H.P.); mslee@konyang.ac.kr (M.-S.L.); 2 Onehealth Research Laboratory, College of Medicine, Konyang University, Daejeon 35365, Korea

**Keywords:** public health, nonpharmaceutical measure, COVID-19, SARS-CoV-2, influenza, parainfluenza, respiratory syncytial virus, rhinovirus, adenovirus, systematic review

## Abstract

Many countries have implemented public health and social measures (PHSMs) to control the spread of severe acute respiratory syndrome coronavirus-2 (SARS-CoV-2). Although the PHSMs are targeted at SARS-CoV-2 transmission control, they directly or indirectly impact the epidemiology of different respiratory viral diseases. The purpose of this study was to investigate the collateral impact of PHSMs used during the coronavirus disease 2019 (COVID-19) pandemic on the epidemiology of other respiratory viruses, including influenza, parainfluenza, respiratory syncytial virus, rhinovirus, and adenovirus infections. We conducted a systematic review of the published literature on changes in the incidence of respiratory viral diseases and detection rates of the respiratory viruses during COVID-19 pandemic, lasting from 2020–2021, published between December 2019 and March 2022 in *PubMed, Embase*, and *Cochrane Library* databases. We identified an overall decrease of 23–94% in the incidence of respiratory viral diseases and a decrease of 0–98% in the detection of the viruses. Our study suggests that the PHSMs implemented during COVID-19 pandemic reduced the incidence of respiratory viral diseases and transmission of respiratory viruses. At the time of this study, and as governments relax PHSMs, public health authorities should prepare for a probable increase in the burden of respiratory viral diseases.

## 1. Introduction

Respiratory virus infections are common in people of all ages [1], and acute respiratory infections are mainly viral in origin [2]. It causes secondary bacterial pneumonia, which is the most important clinical complication and has considerable burden on healthcare systems [2,3]. The options for the available intervention to reduce the public health impact of respiratory virus epidemics are vaccines, antivirals, and public health and social measures (PHSMs). PHSMs include the use of face masks, regular hand-sanitizing and handwashing, quarantine and isolation, social distancing, and avoiding gatherings [4,5,6]. The effectiveness of any one of these measures alone is likely to be limited. However, the combination of multiple interventions has been reported to substantially affect the transmission of various diseases [7,8,9,10,11,12].

Coronavirus disease 2019 (COVID-19) is an ongoing pandemic affecting healthcare systems worldwide due to the high associated mortality rate [13]. Since the start of the COVID-19 pandemic, PHSMs have been essential for reducing the transmission of severe acute respiratory syndrome coronavirus-2 (SARS-CoV-2) [4]. Even after the approval of COVID-19 vaccines, PHSMs are still the major strategies used to control the COVID-19 pandemic [14,15,16]. Empirical evidence has shown that these control measures have been effective in preventing the spread of SARS-CoV-2 and in reducing the incidence of COVID-19 [17,18]. Though these behavioral changes are aimed at reducing SARS-CoV-2 transmission, they are likely to have consequences on the epidemiology of other respiratory viruses.

In this review, we focused on five viruses that are the major causes of acute respiratory disease, including influenza, parainfluenza, rhinovirus, adenovirus, and respiratory syncytial virus (RSV) [19]. These viruses share a relatively short incubation period (1–4 days) and a person-to-person mode of transmission. Furthermore, the transmission is direct by infective droplet nuclei or indirect by hand transfer of contaminated secretions to the nose or eyes [20]. These viruses are also the major causes of lower respiratory tract infections, which are the leading causes of morbidity and mortality among children and lead to a substantial public health problem [21]. The impact of PHSMs implemented during the COVID-19 pandemic on various respiratory viral diseases is unclear. In this study, we assessed the changes in the respiratory virus activity including the incidence of respiratory viral diseases and detection rate of the respiratory virus during COVID-19 pandemic 2020–2021 through a systematic review of the literature.

## 2. Materials and Methods

**Figure 1:**
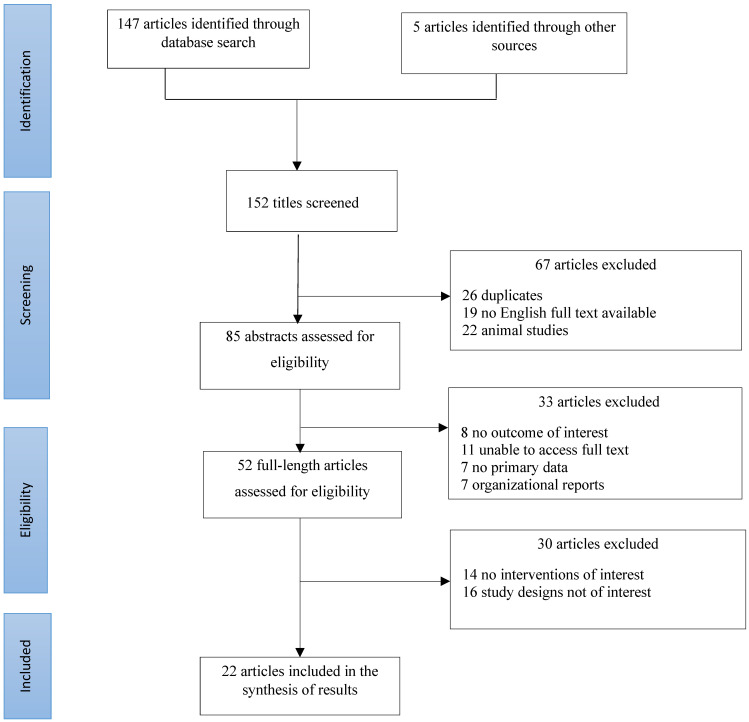
PRISMA flowchart of the search strategy, inclusion and exclusion screening, and accepted studies of the review of changes in the incidence of respiratory viral infections during the COVID-19 pandemic 2020–2021.

We used the Preferred Reporting Items for Systematic Reviews (PRISMA) guidelines for this review (Figure 1) [22]. We developed a review protocol that described the article selection criteria, search strategy, and data extraction procedures. Articles for the systematic review were sourced from *PubMed, Embase*, and *Cochrane Library*. Furthermore, we collected additional published studies from secondary references of included studies and other relevant studies. Accessible peer-reviewed, original research articles with full introduction, methods, results, and discussion sections in English, published between 10 December 2019 and 30 March 2022, were eligible for inclusion.

The search terms used in this study were identified from relevant research articles. The search terms included a combination of Term–1, Term–2, Term–3, and Term–4. Term–1 included COVID-19 or 2019 novel coronavirus disease or COVID19 or COVID-19 pandemic or COVID-19 virus disease or coronavirus disease 2019 or coronavirus disease-19 or 2019-ncov disease. Term–2 included damage or delay or decrease or disrupt or postpone or negative or cancel or benefit or positive or increase. Term–3 included prevalence or incidence or attack rate or diagnosis or screening or care or treatment or positivity rate or cost. Term–4 included infectious diseases or communicable diseases or transmissible disease or respiratory viral diseases or influenza or parainfluenza or rhinoviruses or adenoviruses or respiratory syncytial virus.

We excluded articles if they did not investigate any COVID-19-related impact or were animal studies, editorials, reviews, or commentaries without primary data or peer review. Furthermore, articles without a clearly stated baseline value for impact quantification were excluded. Two independent researchers (A.C. and H.P.) screened article titles and abstracts and assessed full-text articles for eligibility. Two other reviewers (S.R. and M.-S.L.) solved any disagreements between the two reviewers. Duplicates of the searched articles were removed.

Our initial database search identified 147 titles that were screened. We further scrutinized other sources and identified an additional five studies that were publicly available but were not detected by our search. After a thorough review of the identified articles, 22 articles were used in this study. Data extracted included geographical location, the focus of interest, the timing of intervention implementation, population, methodology, and key findings including the reported arithmetic size of impacts (increase or decrease) and the corresponding confidence intervals or the ranges. In cases where the confidence intervals were not reported, we reported the numeric value of the impact size from the finding.

## 3. Results

We identified a total of 22 studies that reported the impact of COVID-19 pandemic on the activity of respiratory viruses including influenza (Table 1), parainfluenza (Table 2), RSV (Table 3), rhinovirus (Table 4), and adenovirus (Table 5).

### 3.1. Influenza

In New Zealand, there was a 67.7% reduction in influenza incidence during the implementation of PHSMs [23]. Similarly, in Mexico, the estimated daily number of influenza cases significantly decreased by 76% in outpatient clinics and public hospitals during the lockdown period (March to May 2020) compared to that in the same period in 2019 [24]. Throughout the implementation of COVID-19 control measures, facility-based surveillance revealed a decline in influenza virus detection during the COVID-19 pandemic period compared with previous years (12.7% versus 4.4%) [25].

In the U.S., the incidence of influenza, which circulated in early 2020, was reduced by more than 60% during the first 10 weeks following implementation of PHSMs [26]. A research in Singapore reported a 76% decline in influenza activity, suggesting that the measures taken for COVID-19 were effective in reducing the spread of other viral respiratory diseases [27].

In Taiwan, a study showed that the infection rates of influenza were lower in 2020 than in the previous year [28]. This observation correlated with the implementation of PHSMs against COVID-19. Similarly, in Hong Kong, influenza transmission declined substantially after the implementation of social distancing measures. The authors observed a 44% (95% CI: 34–53%) reduction in community transmissibility from an estimated *R*_t_ of 1.28 (95% CI: 1.26–1.30) before the start of school closures (which was part of the PHSMs) to 0.72 (95% CI: 0.70–0.74) during the closure weeks [29]. A study in Korea also reported that during the period of enforced social distancing from weeks 9–17 of 2020, the number of hospitalizations due to influenza cases was 11.9–26.9-fold lower than that in the previous year. The study concluded that the PHSMs substantially decreased the seasonal influenza cases and activity [30]. The PHSM-implemented period was associated with a sharp and sustained decline in influenza-detection rates, from a zero detection rate out of 163,296 tests during weeks 23–40 of 2020 to 26.1% case-detection rate in the same period in 2015–2019 [31].

**Table 1:** Summary of influenza-related articles included in the systematic literature review.

Author, Year, and Location of Study	Study Details	Main Findings
Cowling et al., 2020; China [29]	Using telephone surveys conducted on 20–23 January, 11–14 February, and 10–13 March 2020, data on 715 laboratory-confirmed COVID-19 cases were obtained from the Hong Kong Center for Health Protection. The authors estimated the daily effective reproduction number (*R*_t_) for COVID-19 and influenza to estimate changes in transmissibility over time.	Influenza transmission declined after the implementation of PHSMs, with a 44% (95% CI: 34–53%) reduction in transmissibility in the community from an estimated *R*_t_ of 1.28 (95% CI: 1.26–1.30) before the start of school closure to 0.72 (0.70–0.74) during the closure weeks.
Soo et al., 2020; Singapore [27]	Indicators of influenza activity in 2020 before and after COVID-19 PHSMs with the corresponding indicators from 3 preceding years were compared.	The percentage of influenza positivity decreased by 64%, and the estimated daily number of influenza cases decreased by 76% in weeks 5–9 of 2020 compared with the preceding years.
Tempia et al., 2020; South Africa [25]	The authors assessed the detection of influenza and RSV through facility-based syndromic surveillance of adults and children with mild or severe respiratory illness (SRI) from January to October 2020 and compared this with surveillance data from 2013 to 2019.	A decrease in influenza-detection rate was recorded. From 2013 to 2019, there was a 12.7% and 5.2% influenza-detection rate for specimens from ILI and SRI patients, respectively. Meanwhile, in 2020, a corresponding detection rate of 4.4% and 0.8% influenza-detection rate of specimens from ILI and SRI cases was recorded, respectively.
Arellanos-Soto et al., 2021; Mexico [24]	Analysis of sentinel surveillance data on influenza-like illnesses (ILI) evaluating whether the influenza trends in the 2019–2020 season were different before and after the implementation of COVID-19 national control protocol.	The average number of influenza cases during the COVID-19 PHSM implementation was significantly different from that during the previous two influenza seasons (587 vs. 357). The percentage of influenza cases decreased by 64%, and the estimated daily number of influenza cases decreased by 76% in week 20 of season 2019–2020 compared with the preceding years.
Huang et al., 2021; New Zealand [23]	Using multiple surveillance systems from May to September 2020, the authors observed trends of influenza and other respiratory viral infections in 2020.	From 1 January to 31 July 2020, a total of 291 influenza hospitalizations were as follows: pre-lockdown 238 (81.8%), lockdown 33 (11.3%), and post-lockdown 15 (5.2%).
Kim et al., 2021; South Korea [30]	The authors analyzed changes in sample positivity by respiratory viruses after PHSMs.	Compared with the pre-PHSMs period, the positive rates of RSV and influenza decreased significantly to 19% and 6% and 23% and 6% of the predicted value.
Lee et al., 2021; South Korea [28]	National influenza surveillance data were compared over seven sequential seasons in April 2020.	A decrease in seasonal influenza cases, hospitalization, and activity was seen after the implementation of PHSMs. The peak activity was lower in 2019/2020, with 49.8 ILIs/1000 visits, than in other seasons showing values of 71.9 to 86.2 ILIs/1000 visits. ILI activity also decreased during weeks 9 to 17 (−12 ILIs/1000 visits on average; 95% CI: −18 ILIs/1000 visits to −6 ILIs/1000 visits).
Qi, et al., 2021; United States [26]	An absolute humidity-driven susceptible-infectious-recovered-susceptible (SIRS) model was used to quantify the reduction in influenza incidence and transmission after implementation of PHSMs in 2020.	The incidence of influenza, which circulated in early 2020, was reduced by more than 60% in the United States during the first 10 weeks following implementation of PHSMs.
El-Heneidy et al., 2022; Australia. [31]	Weekly counts of influenza and other respiratory diseases from a Queensland laboratory network were obtained for the year 2020 and compared with averaged counts from 2015 to 2019.	PHSMs were associated with a sharp and sustained decline in influenza, whereas during the typical annual influenza season (weeks 23–40), no cases were detected from 163,296 tests compared with an average of 26.1% (11,844/45,396) of tests positive in 2015–2019.

### 3.2. Parainfluenza

An 85% reduction in hospitalizations due to the parainfluenza (PIV) virus cases in children in 2020 was recorded in Hong Kong after the implementation of COVID-19 interventions as compared to previous years [32]. In 2020, during the PHSMs implementation period, a study reported that there were no cases of parainfluenza detected in the 70,618 tests performed compared with the 4.2% (1495/35,754) average detection rate in the 2015–2019 seasons [31]. In addition, children were likely more affected with the monthly incidence among children aged 0–4 years (120 per 100,000 children) being six times higher than the monthly incidence (21 per 100,000 children) during the previous 10 years [33]. In China, the incidence of PIV1, PIV2, and PIV3 in 2020 was significantly decreased compared to that in 2019 [34]. However, as the restrictions were relaxed in September 2021, a high epidemic peak of parainfluenza was observed [35].

**Table 2:** Summary of parainfluenza-related articles included in the systematic literature review.

Author, Year, and Location of Study	Study Details	Main Findings
Liu et al., 2021; China [34]	Respiratory specimens were obtained from children with lower respiratory tract infections at Children’s Hospital of Fudan University, and data were analyzed and compared between the year 2020 (COVID-19 pandemic) and 2019 (before COVID-19 pandemic).	Parainfluenza viruses were detected in 460/2507 (18.35%) specimens in 2020, which was significantly lower than that in 2019 (1072/4600, 23.30%).
El-Heneidy et al., 2022; Australia [31]	Weekly counts of parainfluenza and other respiratory diseases from a Queensland laboratory network were obtained for the year 2020 and compared with averaged counts from 2015 to 2019.	The number of positive tests for influenza decreased to zero in 2020 compared to 1053 in 2015–2019 following the introduction of PHSMs.
Kuitunen et al., 2022; Finland [33]	A nationwide register-based retrospective epidemiologic surveillance study was conducted from January 2012 to December 2021.	The monthly parainfluenza incidence among children aged 0–4 years was six times higher than that in the previous years. As the restrictions were relaxed in September 2021, a high epidemic peak of parainfluenza was recorded after relatively low levels.

### 3.3. Respiratory Syncytial Virus

In South Korea, compared to the pre-PHSMs period, the positive rates of RSV decreased from 19% to 6% in the PHSMs implemented period [36]. In Japan, the average number of monthly RSV case notifications in 2020 decreased by approximately 85% compared to those in the preceding 6 years (2014–2019) [37]. In Canada, the percent positive rate for RSV dropped by 0.02% of that of pre-pandemic (August 2014–March 2020) levels [38]. Similarly, a 98% reduction in RSV cases was observed in western Australian children through the winter season in 2020 during the implementation of PHSMs [39]. Contrarily, in China, the positive rate of RSV in 2020 was significantly higher than that in 2019 (9.35% vs. 6.31%) [34] with the 2020 peak month, December, contributing to 75% of RSV-positive admissions, 2.5-times higher than the previous 2019 peak [34]. In Rome, during the epidemic period, the total number of case-patients of RSV dropped from 726 in 2018–2019 to 689 cases in 2019–2020 [40].

**Table 3:** Summary of RSV-related articles included in the systematic literature review.

Author, Year, and Location of Study	Study Details	Findings
Groves et al., 2021; Canada [38]	Epidemiologic data were obtained from the Canadian Respiratory Virus Detection Surveillance System. Weekly data from the week ending 30 August 2014 until the week ending 13 March 2021 were analyzed.	The percent positive rates for RSV dropped by 0.02% in the post-pandemic period compared to that in the pre-pandemic levels.
Kim et al., 2021; South Korea [36]	The Korean influenza and respiratory virus-monitoring system database was used. From January 2016 through January 2021, the weekly positive rate of respiratory viruses and the weekly number of hospitalizations with acute respiratory infections were investigated.	Compared with the pre-PHSMs period, the positive rates of RSV decreased significantly from 19% to 6% in the PHSMs implemented period.
Wagatsuma et al., Japan 2021 [37]	The monthly number of RSV cases per sentinel site in 2020 was compared with the average of the corresponding period in the previous 6 years using a monthly paired *t*-test.	The average number of monthly RSV case notifications in 2020 decreased by approximately 85% compared to that in the preceding 6 years (2014–2019).
Yeoh et al., 2021; Australia [39]	Laboratory data were prospectively collected as part of routine regional public health surveillance and analyzed weekly from 1 January 2012 to 30 August 2020.	Overall, 98.0% reductions in RSV in children through winter 2020 compared to previous seasons (2012–2019).
Vittucci et al., 2021; Italy [40]	A retrospective analysis of nasopharyngeal samples of all patients (0–18 years old) admitted with respiratory symptoms in a large Italian tertiary hospital during the last three seasons from 2018 to 2021 was conducted.	There was a decrease in RSV cases from 726 in 2018–2019 to 689 in 2019–2020 during the COVID-19 pandemic.
El-Heneidy et al., 2022; Australia [31]	Weekly counts of RSV and other respiratory diseases from a Queensland laboratory network were obtained for the year 2020 and compared with averaged counts from 2015 to 2019.	RSV-detection rates decreased in weeks 39–47 after the implementation of PHSMs but increased to 5.6% (562/10,078) in weeks 48–52 in 2020 from 2.9% (150/5018) in 2015–2019.
Ye et al., 2022; China [34]	Epidemiologic characteristics of common childhood respiratory viruses in 2020 (after the pandemic) compared with 2019 (before the pandemic) were examined.	The positive rate of RSV in 2020 was higher than that in 2019 (9.35% vs. 6.31%).

### 3.4. Rhinovirus

In South Korea, after social distancing measures were implemented, there was a significant decrease in the monthly mean rhinovirus incidence rate relative to the pre-pandemic period (–60.4% to –93.8%) [41]. The impact of the restrictions was mostly observed among children aged 0–4 years of age in weeks 14–22 in 2020. Strict restrictions temporarily interrupted the circulation of rhinovirus in spring 2020. However, rhinovirus incidence returned to normal levels soon after the strict restrictions were relaxed [42].

**Table 4:** Summary of rhinovirus-related articles included in the systematic literature review.

Author, Year, and Location of Study	Study Details	Findings
Kuitunen et al., 2021; Finland [42]	Rhinovirus epidemiology in children during the pandemic was analyzed using data from the Finnish Infectious Disease Register.	There was a 22.6% decrease in rhinovirus incidence during the COVID-19 pandemic. The impact of the PHSMs was mostly seen among children aged 0–4 years of age in weeks 14–22 in 2020.
Park et al., 2021; South Korea [41]	National surveillance data were used to compare the incidence of respiratory viruses during 2016–2019 vs. 2020.	In South Korea, after social distancing measures were implemented, there was a significant decrease in monthly mean rhinovirus incidence rate relative to the pre-pandemic period (–60.4% to –93.8%).

### 3.5. Adenovirus

In a study in China, the positive rate of adenovirus in 2020 was significantly lower than the level in 2019 (−2.69%) [43]. In Japan, during the implementation of PHSMs, there was a 52% increase in sample positivity rate for adenoviruses, and a change in virus diversity was also observed [44].

**Table 5:** Summary of adenovirus-related articles included in the systematic literature review.

Author, Year, and Location of Study	Study Details	Findings
Li et al., 2021; China [43]	Data on tests of adenovirus from electronic healthcare records of Children’s Hospital of Zhejiang University School of Medicine were extracted during the COVID-19 pandemic (January–December 2020) and were compared with those in 2019 during the same period.	The positive detection rate of adenovirus in 2020 was 2.69% lower than the level in 2019.
Nagakubo et al., 2022; Japan [44]	Nasopharyngeal swab samples from 3249 patients who visited the Yamanashi Central Hospital in Japan from 1 March 2020 to 28 February 2021 were used to determine the adenovirus positive rate.	During the implementation of PHSMs, there was a 52% increase in sample positive rate for adenoviruses; a change in virus diversity was also observed.

## 4. Discussion

Understanding the possible influence of PHSMs during COVID-19 pandemic on the incidence of other respiratory viral diseases gives a broader picture of the public health impact of the COVID-19 pandemic. This review highlights the collateral impact of COVID-19 and its control measures on respiratory viral diseases.

Influenza was one of the common respiratory infectious diseases reported to be greatly impacted by the PHSMs. In response to the COVID-19 pandemic, PHSMs also reduced the spread of influenza in 2020, characterized by an initial increase in influenza testing in some countries followed by a decline in the detection of seasonal influenza coinciding with the implementation of PHSMs for COVID-19 control [45]. The continuous practice of PHSMs against COVID-19 resulted in an overall reduction in influenza infections during the winter months compared with previous seasons. This continuous effort to contain COVID-19 using PHSMs saved lives directly by reducing the incidence of seasonal influenza and SARS-CoV-2 as well as by reducing the influenza-related burden on the healthcare system [7,45]. This implies that continuous implementation of PHSMs in the post-COVID-19 era is likely to benefit control programs for influenza and other respiratory viral diseases worldwide.

Apart from reports about influenza, other findings showed decreasing changes in the epidemic trends of parainfluenza, RSV, rhinovirus, and adenovirus. For the parainfluenza and RSV, the incidence pattern, which has significant reductions in the number of cases during the implementation of COVID-19 control measures with surges after the control measures were relaxed, was similarly observed. These results may suggest that the series of preventive and control measures against SARS-CoV-2 were also effective in stopping the spread of RSV and parainfluenza viruses but did not continuously sustain these viruses at low levels.

Rhinovirus and adenovirus infections also decreased during the strict implementation of PHSMs with rhinovirus surges in children after school reopening. This rebound of incidence after opening schools was observed in a previous study of influenza [46].

While PHSMs are effective in mitigating the epidemic, there are some aftereffects to be considered in the post-COVID-19 era. Long-term effects of the implementation of PHSMs may be substantial especially in low- and middle-income settings where public health resources are limited. Prolonged PHSMs could increase health inequalities in the short and long term, penalizing more vulnerable communities with negative effects on both physical and mental health. For example, quarantine of people exposed to an infectious disease is associated with negative psychological effects, including post-traumatic stress symptoms, which may be long-lasting [47]. Social isolation is associated with an increase in mortality [47]. Longer periods of social distancing may have similar effects.

As much as COVID-19 has dominated the landscape of respiratory viral infections since December 2019, it is important for clinicians and public health practitioners to recognize that the return of other respiratory viral infections may be rapid and significant when COVID-19 containment measures are removed [48]. Therefore, public health authorities to be alert for upcoming burden of respiratory viral diseases.

Our study has several strengths. First, we systematically evaluated and reported the evidence accumulated between December 2019 and March 2022, which is of practical value for policy makers and researchers. Second, we explored the quantitative impact of PHSMs on different respiratory viruses in different countries, which is important for summarizing the effect of the COVID-19 pandemic. Third, the study revealed some evidence gaps that could help future researchers. However, our study also has some limitations. The risk of publication bias cannot be ruled out, as some published studies related to our subject of interest could have been missed. In addition, a significant degree of heterogeneity of PHSMs was noted across studies, making it difficult to compare the effect-sizes of PHSMSs in different settings.

## 5. Conclusions

Our study suggested that the implemented PHSMs during COVID-19 pandemic had a collateral effect on the reduced incidence of respiratory viral infections. As the COVID-19 pandemic continues, further research is needed to generate more evidence on the collateral impact of COVID-19 on different aspects of the healthcare system, particularly from developing regions of the world. At the time of this study, as governments relax PHSMs, public health authorities should prepare for the upcoming burden of respiratory viral diseases.

## Data Availability

All the data used in this study are available online as published articles.
